# Income inequality and healthcare utilization of the older adults-based on a study in three provinces and six cities in China

**DOI:** 10.3389/fpubh.2024.1435162

**Published:** 2024-07-24

**Authors:** Zhang Chi, Hai Lun, Jiaxin Ma, Yaping Zhou

**Affiliations:** ^1^School of Philosophy and Sociology, Lanzhou University, Lanzhou, China; ^2^School of Public Policy and Administration, Xi'an Jiaotong University, Xi'an, China

**Keywords:** older adults, healthcare utilization, income inequality, equity, public transfer

## Abstract

**Purpose:**

The objective of this study is to gain a more nuanced understanding of the specific impact of income inequality on the utilization of healthcare services for older adults. Additionally, the study aims to elucidate the moderating and mediating roles of public transfer income and psychological health in this context.

**Methods:**

A systematic examination of the impact of income inequality on healthcare utilization among older adults was conducted through field questionnaire surveys in six cities across three major geographical regions (West, Central, and East). The analysis employed baseline regression, as well as mediating and moderating effect tests.

**Results:**

First, there is a negative relationship between income inequality and the use of therapeutic healthcare services (β_1_ = −0.484, *P* < 0.01) and preventive healthcare services (β_2_ = −0.576, *P* < 0.01) by older adults. This relationship is more pronounced in the low- and medium-income groups as well as in the western region. The mediating effect of psychological state is significant (β_3_ = −0.331, *P* < 0.05, β_4_ = −0.331, *P* < 0.05). Public transfer income plays a significant role in regulation. The moderating effect of public transfer income on therapeutic services was more significant in low-income groups (β_5_ = 0.821, *P* < 0.01). The moderating effect of public transfer income on preventive services was more significant in middle-income groups (β_6_ = 0.833, *P* < 0.01).

**Conclusion:**

The study clearly demonstrates a significant negative correlation between income inequality and the utilization of healthcare services by older adults. Furthermore, the study reveals that this relationship is particularly pronounced among older adults in low- and medium-income and Western regions. This detailed analysis of regional and income level heterogeneity is of particular value in this field of research. Secondly, this study attempts to integrate the two pivotal dimensions of public transfer income and psychological state for the first time, elucidating their moderating and mediating roles in this relationship. The findings indicate that public transfer income serves as a moderating factor, exerting a notable “reordering effect” on income inequality and resulting in a “deprivation effect.” Such factors may impede the utilization of medical services, potentially influencing the psychological state of older adults.

## Background

Currently, China is implementing a national policy focused on active aging in conjunction with the objective of promoting a healthy China. According to data from the seventh national population census, there are 264.02 million individuals aged 60 and above in China, which represents 18.70% of the total population. Of this population, 190.64 million people are aged 65 and above, accounting for 13.50% of the total population (1). Consequently, the phenomenon of population aging has been intensifying in China at this stage. The increasing number of older adults individuals in China has resulted in a significant healthcare burden as well as posing a challenge to social and economic development (2, 3). A report from the China Association for Older Adults indicates that over 180 million individuals aged 60 or above suffer from chronic diseases. The provision of healthcare services for this demographic is thus of paramount importance for individuals, families, and society (4). Nevertheless, there remain significant practical issues in China, These include the inequitable allocation of healthcare resources between urban and rural regions, the financial strains imposed on healthcare care, and the incomplete hierarchical diagnosis and treatment system (5, 6).

China is currently engaged in a process of reforming its distribution system with the objective of promoting common prosperity in a manner that is people-centered. The issue of realizing this goal, particularly for older adults, is of great urgency (7). Since the implementation of reform and opening-up policies, there has been a notable improvement in the wellbeing of older adults, with pension income demonstrating consistent and sustainable growth over the past 19 periods (8). The income of older adults has not only surpassed the economic growth rate but also outpaced the income growth of urban residents during the same period. Consequently, the income gap within this cohort has become increasingly pronounced as their earnings rise gradually (9). The income disparity among older adults in China is currently substantial, with indications that it is increasing. This poses a risk of deterioration in income distribution for this demographic, which has become a pressing concern for society (10).

In recent years, the impact of the COVID-19 pandemic has led to an increase in the health needs of older adults in China. However, they have also exhibited a tendency to endure minor illnesses and to bear major illnesses. This unique phenomenon may be related to the widening income gap among older adults in our country. Government transfer payments represent an important means of regulating the distribution of income among older adults at the micro-individual level, specifically in the secondary distribution of transfer income from the government. It is important to note that this transfer payment plays a significant role at the micro-individual level, specifically in the secondary distribution of transfer income from the government. This distribution primarily encompasses old-age pensions (or retirement pensions), low-income insurance, a variety of cash subsidies, and other items. These items can be used to reflect the degree of residents' ability to obtain social security and public services (11). Transfer payment is an automatic stabilizer of the economy (12), which can reduce the income gap between groups and achieve income redistribution through the government's direct transfer of wealth to the disadvantaged older adult group, which in turn affects consumption demand. In this context, it is of great theoretical value and practical significance to study the intrinsic association between healthcare services for older adults in China and income inequality and government transfer payments.

## Theoretical and research framework

As income inequality gets worse, people are paying more attention to how it affects healthcare (13). In particular, academics are interested in the impact of income disparity on healthcare service utilization. The healthcare services market for older adults exhibits two types of resource mismatches. First, high-income older adults with minor illnesses pay a significant premium for the services of skilled doctors, which diverts skilled doctors' time and energy from addressing common illnesses (14). Secondly, some older adults with serious illnesses and low incomes are unable to access treatment from skilled doctors, which can result in misdiagnosis and the abandonment of treatment. This leads to a higher likelihood of receiving better quality care for patients with minor illnesses compared to those with serious illnesses. The resource mismatch is a consequence of income differences and the specifics of the healthcare market (15). In the meantime, current research on income level variations focuses on the entire population. This study, however, examines income groups in a particular geographic area, as older adults' spending habits are influenced by their surroundings (16, 17). Existing studies mainly concentrate on the utilization of therapeutic healthcare services. However, due to the “Healthy China 2030” initiative, the usage of preventive healthcare services by older adults in China has notably increased (18, 19). The aforementioned studies do not differentiate between the two. Consequently, this study represents a novel approach by categorizing healthcare service utilization for older adults into preventive and therapeutic categories and conducting distinct analyses of income inequality and service utilization (20). The objective of this study is to categorize the utilization of healthcare services by older adults as either curative or preventive and to examine the impact of income inequality on each type of healthcare services use (21).

Public transfers improve social income inequality through income redistribution, thereby affecting the utilization of healthcare services for the older adults. Existing related literature can be broadly divided into two categories, namely, the impact of public transfers on the income distribution situation and the impact of public transfers on the utilization of healthcare services for the older adults (11, 22). In terms of public transfers affecting income distribution, Qingwang et al. (23) found that government transfer expenditures have a narrowing effect on income inequality among the older adults in China and have a strong reordering effect on the income distribution of the older adults (24). The absolute income hypothesis (Keynes) argues that high-income older adults have a lower propensity for healthcare services, and the widening income gap will reduce the consumption rate of society as a whole, so income inequality inhibits healthcare utilization (25). The absolute and relative poverty theories suggest that in a society with income inequality, relative poverty may be more pronounced, resulting in low-income older adults being disadvantaged socially, economically, and in terms of health (26).

The social determinism theory posits that an individual's health status is contingent upon their social and economic milieu. In a society where income inequality is prevalent, older adults with low incomes may be more susceptible to health risk factors, such as substandard housing, inadequate nutrition, and high-stress living environments (27). Older adults are frequently retired and lacks a consistent source of income. In societies where income inequality is prevalent, older adults may lack the financial resources to afford healthcare, particularly if they lack adequate healthcare coverage (23). In a society where income inequality is prevalent, healthcare resources may be disproportionately allocated to those who can afford them, creating challenges for older adults in accessing quality healthcare (28).

Moral hazard theory theorizes that when individuals are covered by insurance, they may increase risky behavior (29). In contrast, however, when individuals lack adequate insurance coverage, they may forgo necessary healthcare services to reduce costs. In a society with income inequality, low-income older adults may refrain from seeking healthcare assistance due to a lack of sufficient insurance coverage.

Based on the above analysis, while this study examines the healthcare utilization of the older adults from a micro perspective, the concern is how the healthcare utilization of individual older adults changes with income inequality. The following hypotheses are proposed:

Hypothesis A: income inequality discourages older adults healthcare services utilization.

**Figure 1:**
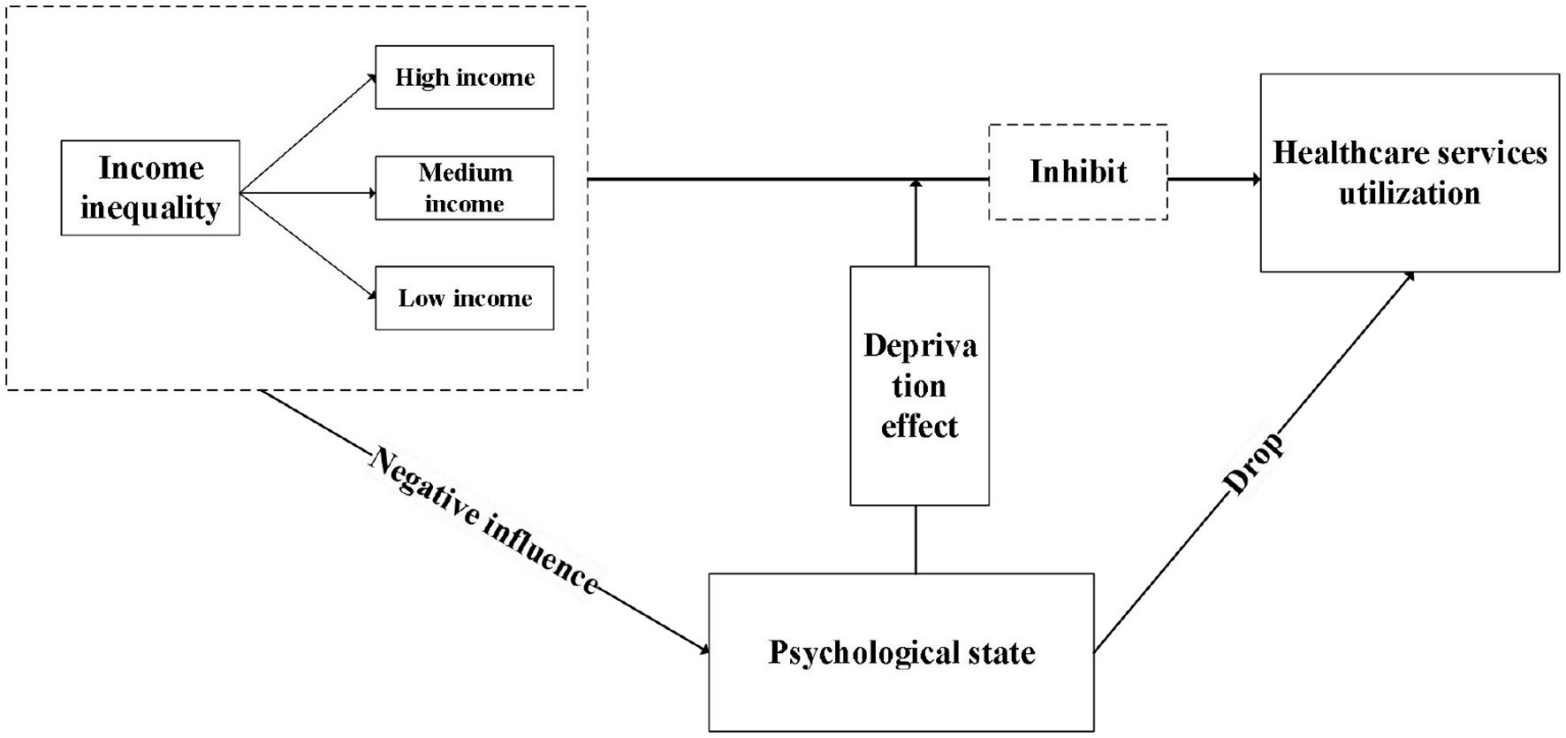
The mediating role of psychological state.

The impact of income inequality on the psychological health of older adults and its subsequent effect on healthcare utilization is a complex and interrelated process. Social comparison theory posits that individuals tend to compare themselves to others in their social environment (30). When older adults observe economic disparities between themselves and others, they may experience psychological dissatisfaction, frustration, or low self-esteem, which may affect their psychological health. Chronic income inequality may result in low-income groups in society feeling marginalized and isolated (31). This sentiment may give rise to a loss of confidence in the healthcare system, which may result in a reluctance or delay in seeking healthcare services (32, 33). Healthcare utilization among older adults with lower psychological health is significantly lower than among older adults with higher psychological health. Income inequality exacerbates this psychological health effect on healthcare service utilization (34). Consequently, the following hypothesis is put forth in this study (the framework is displayed in Figure 1):

Hypothesis B: the impact of income inequality on the utilization of healthcare services can be influenced by affecting the psychological state of older adults.

Public transfers of income, often referred to as non-labor income, such as social security benefits, pensions, health insurance subsidies, etc., provided by governments or other public institutions. Such transfers serve as an important economic safety net in many countries, especially for those with low or no stable sources of income (35). As a significant component of the income of older adults, public transfers can not only directly enhance the wealth of the population and stimulate consumption, but also indirectly contribute to an increase in the level of social welfare through redistribution. Public transfers are distinguished by their predictability and durability and stability compared to other forms of short-term income (36). For example, old-age pensions and retirement income can provide a continuous and stable source of income for the older adults population; and low-income subsidies can provide the most basic livelihood security for eligible older adults (37). Low-income older adults are the main beneficiary group of public transfer income, and the promotion of social equity is one of the important goals of the transfer system. The widening of the income gap has been shown to affect social trust and the sense of social fairness, as well as to have an adverse effect on the demand for healthcare services (38). However, the implementation of a sound transfer payment system has the potential to improve the sense of social fairness among older adults. On the one hand, a lack of fairness makes it difficult for older adults to have stable expectations for the future, which in turn leads them to increase their savings in order to cope with potential risks in the future (39). Therefore, public transfer income can alleviate the inhibitory effect of income inequality on the utilization of healthcare services by the older adults. This study proposes the following hypothesis:

Hypothesis C: public transfer revenues can play a positive moderating role in the way income inequality affects healthcare utilization.

**Figure 2:**
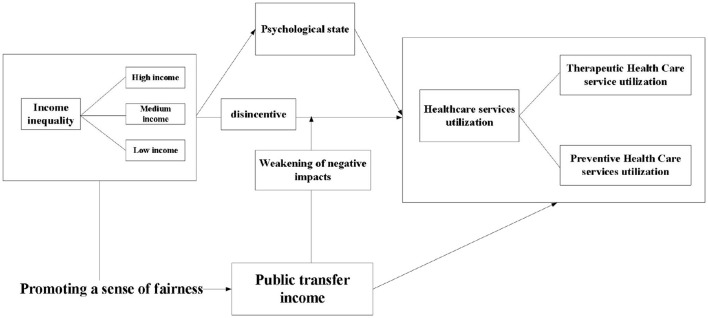
Research framework diagram.

This framework (Figure 2) diagram shows how public transfer revenue positively moderates the effect of income inequality on healthcare utilization among older adults. Income inequality may lead to less frequent utilization of healthcare services by older adults, but public transfer revenues can mitigate this effect.

Of these, income inequality serves as the primary independent variable, directly affecting healthcare utilization among older adults. Additionally, it may indirectly affect healthcare utilization by influencing psychological health. Psychological health, in turn, may be affected by income inequality, potentially influencing healthcare utilization among older adults (40). The older adults were classified into three categories: low-income, medium-income, and high-income. The income inequality variable in Figure 2 represents the starting point of our study and illustrates the distribution of income among the various segments of society. This variable directly affects healthcare utilization among older adults. Additionally, it may indirectly affect healthcare utilization by influencing psychological health (41).

The figure illustrates the potential impact of income inequality on healthcare utilization among older adults. In addition to being affected by income inequality in the framework, may itself affect healthcare utilization among the older adults. The moderating variable in the figure is public transfer income (42). This is a moderator that represents the income that the government or society provides to the older adults through some mechanism that mitigates the impact of income inequality on healthcare utilization. The negative arrow from “Income Inequality” to “healthcare utilization among older adults” indicates that income inequality may lead to lower healthcare utilization among older adults (43). Conversely, a positive arrow from “Public Transfer Revenue” to the medium arrow indicates that public transfer revenue helps to mitigate the negative impact of income inequality on healthcare utilization among older adults.

## Data

The research data of this study have relied on the major project of philosophy and social science of the Ministry of Education, the group of 20 teachers and students as investigators. The survey selected the provinces of Shaanxi, Hubei, and Zhejiang, which are located in the western, central, and eastern parts of China, and can better represent the characteristics of the older adult population and the level of socio-economic development in different regions of China. In the selection of cities, Yan'an in the northern part of Shaanxi and Xi'an in the central part were chosen to represent Shaanxi. Jingmen and Wuhan in Hubei were selected to represent Hubei, and Ningbo and Shaoxing were selected to represent Zhejiang. The target respondents are local older adults people over 60 years old, and a total of 1,918 questionnaires are collected. The existing survey sample is already typical and representative. Excluding missing values and invalid questionnaires, and finally 1,882 questionnaires are selected for this study. These data do not involve any personal privacy information and are only used for research purposes. All the surveyed older adults choose not to sign the informed consent form.

The questionnaire data collected by the research is close to the actual situation of the older adults in the research location, and has a certain degree of representativeness. This study uses SPSS 22.0 software to test the reliability of the questionnaire data from the test results can be seen in the questionnaire, Cronbach α system is 0.925, >0.9 judgment standard.

## Variables

The explanatory variable of this article is healthcare utilization of the older adults, and the study categorizes the healthcare utilization of the older adults into two types, which are therapeutic healthcare and preventive healthcare. The core explanatory variable of this article is the degree of income inequality. There are many ways to measure income inequality in current academic circles, and this study chooses the Gini coefficient, which is more widely used and recognized, to measure the degree of income inequality.

Income level is one of the important factors affecting the utilization of healthcare services for the older adults. The income of the older people in the study is mainly their own pension, and we ask the older adults surveyed about their income using the question “Your pension income in the past year.” The level of income of the older adults largely determines the ability and preference of the older adults healthcare service utilization. According to the relative income hypothesis, utilization expenditures depend not only on the absolute level of income but also on the relative income level. We argue that healthcare utilization among the older adults is primarily influenced by their geographic proximity, i.e., the healthcare utilization behavior of the older adults will vary depending on the location of their income distribution in their environment. For this reason, we divided the total sample into three groups according to the level of income of the sample older adults within their geographic area and counted the healthcare service utilization of older adults in different groups (Table 1). Among them, low-income group households were the lowest one-third of the income level ranked in their districts and counties, high-income group households were the highest one-third, and medium -income group households were the medium one-third. As can be seen from Table 1, there is a strong relationship between the utilization of healthcare services by the older adults and the differences in their relative income levels. Influenced by factors such as utilization ability, demand structure and social status, there is heterogeneity in the utilization behavior of healthcare services for the older adults at different income levels. Therefore, in the next analysis of this study, the sample of older adults people will also be grouped according to their income levels (Table 1). Table 1 shows distribution of the surveyed older adults by income distribution.

**Table 1:** Distribution of the surveyed older adults by income distribution.

	**Quorum**	**Percentage**
Lower income	436	23.2%
Medium income	1,006	53.4%
High income	440	23.4%
Aggregate	1,882	100%

In this article, the Gini coefficient is calculated by the following formula:


$$\begin{array}{l} G=1+1/N-2/\left(m\cdot N^{2}\right)\sum_{i=1}^{N}\left(N-i+1\right)y_{i} \end{array}$$

Where, *G* is the Gini coefficient, *N* is the sample size, *m* is the average older adults' income, older adults' income in ascending order, *y*_*i*_ is the income of the *i* older adults. A Gini coefficient of 1 means absolute inequality, while a Gini coefficient of 0 means absolute equality. There are many ways of calculating the Gini Coefficient, and the results may be different for different methods.

The study chose to use the questionnaire “whether or not they had been hospitalized in the past year” to represent therapeutic healthcare services for the older adults, “1 = have therapeutic healthcare services” and “2 = no therapeutic healthcare services.” The questionnaire “whether or not they had a health check-up in the past year” is used to represent preventive healthcare services for the older adults, “1 = have a health check-up” and “2 = no health check-up.”

The study defines the public transfer income explanatory variable as the public transfer income received by the older adults in the year, and the study also defines a dichotomous variable to indicate whether or not the household received public transfer income in the year, with a value of “1” if such income was received, and “0” otherwise. The transfer income in the questionnaire of this study represents public transfer income, which mainly includes old-age pensions (retirement pensions), government subsidies and donations or compensation income, of which government subsidies include low income insurance, subsidies for returning farmland to forests, agricultural subsidies, subsidies for five-guaranteed households, subsidies for special-needs households, pensions for immediate family members of the injured and disaster relief payments, and so on.

The mediator variable is the current psychological state of the older adults, which must be taken into account when reviewing the state of mind of the older adults. This will facilitate a correct perception of healthcare services, thus enabling effective utilization of healthcare services. The specific question is: “How do you feel about your state of psychological?,” and the choices of answers are “1 = very bad,” “2 = not very good,” “3 = generic,” “4 = better,” and “5 = very good.”

The control variables of this study contain six control variables. Control variables were selected with reference to Yang et al. (44). Specifically, they are household registration, the education level of the older adults, the number of children of the older adults, the current health status of the older adults, and the satisfaction of the older adults with health insurance and pension insurance. For the above variables, the Likert five-point method is adopted as the indicator evaluation method, and the options are categorized into five levels from weak to strong as “1, 2, 3, 4, 5,” and the descriptive statistics of all variables are shown in Table 2.

**Table 2:** List of variable specific descriptions.

**Variables**	**Average value**	**(Statistics) standard deviation**
**Healthcare services utilization**		
Therapeutic healthcare services	0.74	0.448
Preventive healthcare services	0.79	0.492
Gini coefficient	0.384	0.062
Household income	50,807	56,463
Public transfer income	0.205	0.361
Receipt of public transfer income (0–1 variable)	0.93	0.431
Psychological state	4.03	0.876
Household registration	1.43	0.504
Educational attainment	2.74	1.587
Number of children	2.46	1.402
Health status	0.79	0.410
Pension insurance satisfaction	3.49	1.189
Satisfaction with healthcare insurance	3.66	1.099

## Results

This study chooses the benchmark model for empirical research, and the model estimation results are shown in Table 3, where the explanatory variables of Model 1 and Model 2 are the therapeutic healthcare service utilization of the older adults, and the explanatory variables of Model 3 and Model 4 are the preventive healthcare service utilization. Models 1 and 3 do not add any control variables, and only regress the Gini coefficient with two different kinds of healthcare service utilization, which serves as a control; Models 2 and 4 add further control variables for regression on this basis.

**Table 3:** Benchmark model regression results.

**Variables**	**Therapeutic healthcare services**		**Preventive healthcare services**	
	**Model 1**	**Model 2**	**Model 3**	**Model 4**
Gini coefficient	−0.325^***^	−0.484^***^	−0.576^**^	−0.739^***^
	(−3.541)	(−4.873)	(−2.246)	(−6.414)
Household income		0.316^***^		0.382^**^
		(3.047)		(2.335)
Public transfer income		0.305^***^		0.412^**^
		(3.552)		(2.347)
Household registration		0.097^**^		0.085^**^
		(2.045)		(2.371)
Educational attainment		0.174		0.136^***^
		(1.237)		(3.962)
Number of children		0.087		0.062
		(1.254)		(1.061)
Health status		−0.213^***^		0.258
		(−3.101)		(1.443)
Pension insurance satisfaction		0.126		0.291^**^
		(1.271)		(3.104)
Satisfaction with healthcare insurance		0.1454^***^		0.204^***^
		(3.338)		(2.915)
Constant term (math.)	15.474	16.831	18.443	18.962
*F*-value	222.434	287.045	201.651	240.382

^*^p < 0.1, ^**^p < 0.05, ^***^p < 0.01.

In Table 3, the estimation results of model (1)-model (4) columns show that the estimated coefficients of the Gini coefficient are all negative and significant at more than 5% level, indicating that income inequality reduces the level of healthcare utilization among the older adults. Thus, Hypothesis 1b is tentatively tested. Specifically, all other things being equal, the Gini coefficient significantly and negatively affects the utilization of curative and preventive healthcare services by the older adults, with a greater impact on preventive healthcare services. In terms of control variables, the results show that the higher the average household income of an older adults person, the higher the probability of therapeutic healthcare service utilization and the probability of preventive healthcare service utilization among the older adults. Public transfer income significantly and positively affects the probability of therapeutic healthcare service utilization and preventive healthcare service utilization, indicating that public transfer income is conducive to promoting healthcare service utilization among the older adults. Education level has almost no significant effect on therapeutic healthcare service utilization of the older adults, while it has a significant positive effect on preventive healthcare service utilization of the older adults. The number of children has no significant effect on the utilization of older adults services. And the level of physical health of the older adults has a negative effect on the utilization of curative healthcare services. Pension insurance satisfaction has a positive and significant effect on preventive healthcare services, but not on curative healthcare services. Health insurance satisfaction has a positive and significant effect on therapeutic and preventive healthcare utilization. The directions of the above estimated coefficients are all as expected. Table 4 shows regression results for older adults with different incomes.

**Table 4:** Regression results for older adults with different incomes.

**Variables**	**Therapeutic healthcare services**			**Preventive healthcare services**		
	**Lower income**	**Medium income**	**High income**	**Lower income**	**Medium income**	**High income**
Gini coefficient	−0.258^***^	−0.364^***^	−0.219	−0.267^**^	−0.507^***^	−0.441
	(−4.148)	(−3.347)	(−1.148)	(−2.351)	(−3.192)	(−5.153)
Household income	0.315^**^	0.441^***^	0.184^**^	0.263^**^	0.481^**^	0.479^**^
	(2.314)	(3.714)	(2.038)	(2.044)	(2.328)	(2.114)
Public transfer income	0.541^***^	0.275^***^	0.088	0.462^***^	0.371^***^	0.013
	(5.661)	(3.148)	(0.162)	(5.147)	(3.029)	(0.035)
Household registration	0.184^***^	0.181^**^	0.207^**^	0.205^**^	0.218^**^	0.234^***^
	(3.014)	(2.155)	(2.141)	(1.991)	(2.013)	(2.874)
Educational attainment	0.014	0.028	0.034^**^	0.021	0.032^**^	0.035^***^
	(0.121)	(0.262)	(1.988)	(0.255)	(2.512)	(4.451)
Number of children	0.068	0.114	0.158	0.127	0.275	0.264
	(0.724)	(0.832)	(0.643)	(0.731)	(1.044)	(1.056)
Health status	−0.262^***^	−0.176^**^	−0.174^**^	0.244	0.287	0.314^***^
	(−2.991)	(−2.247)	(−2.328)	(1.382)	(1.464)	(3.547)
Pension insurance satisfaction	0.279^**^	0.364^***^	0.151	0.184	0.297^***^	0.155
	(2.344)	(3.324)	(1.566)	(1.511)	(3.541)	(1.501)
Satisfaction with healthcare insurance	0.265^**^	0.452^***^	0.163^*^	0.188^*^	0.298^***^	0.147
	(2.384)	(3.047)	(1.802)	(1.758)	(3.475)	(1.441)
Constant term (math.)	14.887	13.904	12.504	11.745	13.874	14.652
*F*-value	147.258	150.441	162.326	204.514	216.910	211.504

^*^p < 0.1, ^**^p < 0.05, ^***^p < 0.01.

In Table 4, the regression coefficients of the Gini coefficient on the utilization of therapeutic healthcare services for older adults people with different incomes are −0.258^***^, −0.364^***^, and −0.219, which indicates that the Gini coefficient has a significant negative effect on the utilization of therapeutic healthcare services for older adults people with low and medium incomes, and does not have a significant effect on older adults people with high incomes. This result implies that as the Gini coefficient increases (i.e., when income inequality increases), therapeutic healthcare service utilization may decrease, especially among older adults in the low- and medium -income levels. The regression coefficients of the Gini coefficient on the utilization of preventive healthcare services for older adults people with different incomes are −0.267^**^, −0.507^***^, and −0.441, which indicates that the Gini coefficient has a significant negative effect on the utilization of preventive healthcare services for low-income and medium -income older adults people, with the greatest effect on medium -income older adults people, and does not have a significant effect on high-income older adults people, which implies that with an increase of the Gini coefficient increases (i.e., when income inequality increases), it decreases the utilization of preventive healthcare services for the low- and medium -income older adults groups.

Household income positively impacts both curative and preventive healthcare for older adults. Public transfer income significantly increases healthcare use for medium and low-income older adults, but not for high-income individuals. Urban residence and higher education levels, particularly among high-income older adults, correlate with greater healthcare utilization. The number of children does not affect healthcare use. Poorer health status generally leads to increased therapeutic healthcare use, while its impact on preventive care varies by income, with high-income older adults showing a greater willingness to invest in preventive measures.

There is a significant positive effect of pension insurance satisfaction on the utilization of curative and therapeutic healthcare services for low-income medium -income older adults, with the greatest effect on medium -income older adults, but the effect on high-income older adults is not significant. There is a significant positive effect of pension insurance satisfaction on preventive healthcare utilization among medium -income older adults, but the effect is not significant for low-income older adults and high-income older adults. There is a significant positive effect of Medicare satisfaction on therapeutic healthcare utilization for all older adults, with the largest effect for medium -income older adults. Medicare satisfaction has a significant positive effect on preventive healthcare utilization among low- and moderate-income older adults, with the greatest effect on medium -income older adults and a non-significant effect on preventive healthcare utilization among high-income older adults.

The regression coefficients of the Gini coefficient on the utilization of curative healthcare services in different regions are −0.541^***^, −0.477^***^, and −0.388^**^, and the regression coefficients of the Gini coefficient on the utilization of preventive services in different regions are −0.558^***^, −0.462^***^, and −0.289^**^, which indicate that the Gini coefficient has a significant negative impact on the utilization of curative healthcare services for the older adults in different regions of the country, as compared to the utilization of preventive healthcare services (Table 5). Preventive healthcare service utilization have significant negative effects, with the greatest effect on the western region and the least effect on the eastern region. This result implies that as income inequality increases, the utilization of curative healthcare services and preventive healthcare services for the older adults may decrease in different regions of the country.

**Table 5:** Regression results for older adults by region.

**Variables**	**Therapeutic healthcare services**			**Preventive healthcare services**		
	**Western part**	**Central section**	**The east**	**Western part**	**Central section**	**The east**
Gini coefficient	−0.541^***^	−0.477^***^	−0.388^**^	−0.558^***^	−0.462^***^	−0.289^**^
	(−4.014)	(−3.582)	(−3.775)	(−4.557)	(−3.874)	(−2.491)
Household income	0.384^**^	0.351^***^	0.301^**^	0.441^**^	0.401^**^	0.365^**^
	(2.311)	(3.545)	(2.264)	(2.405)	(2.247)	(2.337)
Public transfer income	0.104^***^	0.098^*^	0.078	0.091^***^	0.072^**^	0.053
	(3.257)	(1.692)	(1.401)	(3.147)	(2.374)	(0.751)
Household registration	0.164^***^	0.183^**^	0.238^**^	0.184^**^	0.191^**^	0.266^**^
	(3.221)	(2.141)	(2.244)	(2.204)	(2.521)	(2.473)
Educational attainment	0.058^*^	0.046	0.064^*^	0.121^***^	0.133^**^	0.148
	(1.752)	(1.381)	(1.695)	(3.315)	(2.326)	(1.551)
Number of children	0.021	0.041	0.038	0.098	0.087	0.067
	(0.658)	(0.732)	(0.646)	(1.032)	(1.284)	(1.015)
Health status	−0.341^***^	−0.318^***^	−0.447^***^	0.244^*^	0.287^**^	0.325^***^
	(−2.941)	(−3.525)	(−4.147)	(1.738)	(2.416)	(3.774)
Pension insurance satisfaction	0.253^***^	0.287^***^	0.304	0.241^***^	0.287^**^	0.382
	(2.858)	(3.062)	(1.484)	(3.234)	(2.316)	(1.857)
Satisfaction with healthcare insurance	0.121^**^	0.168^***^	0.183	0.158^***^	0.176^**^	0.134
	(2.312)	(3.126)	(1.405)	(3.018)	(2.421)	(1.387)
Constant term (math.)	22.0744	24.506	30.887	20.971	21.887	25.663
*F*-value	197.343	175.377	152.856	137.078	167.497	211.054

^*^p < 0.1, ^**^p < 0.05, ^***^p < 0.01.

Household income has a significant positive effect on both curative and preventive healthcare utilization for all older adults. Public transfer income has a significant positive effect on curative and preventive healthcare utilization for the older adult population in the western region, while it has no significant effect on either type of healthcare utilization for older adults in the eastern region. Household registration has a significant positive effect on both curative and preventive health service utilization among older adults in all regions, with older adults living in urban areas having a much higher health service utilization than those living in rural areas.

There is a significant positive effect of pension insurance satisfaction on the utilization of therapeutic healthcare services and preventive healthcare services for the older adults in the western and central regions, with the greatest effect on the older adults in the central region, but the effect on the older adults in the eastern region is not significant. Healthcare insurance satisfaction has a significant positive effect on the utilization of curative healthcare services and preventive healthcare services of the older adults in the western and central regions, with the greatest effect on the older adults in the central region, but the effect on the older adults in the eastern region is not significant. Table 6 shows mediated effects test.

**Table 6:** Mediated effects test.

**Variables**	**Psychological state**	**Therapeutic healthcare services**		**Psychological state**	**Preventive healthcare services**	
Gini coefficient	−0.457^***^		−0.374^***^	−0.508^***^		−0.426^***^
	(−4.447)		(−2.871)	(−3.834)		(−4.347)
Psychological condition		0.461^***^	0.331^**^		0.496^***^	0.288^**^
		(4.021)	(2.141)		(3.146)	(2.437)
Household income	0.351^***^	0.407^***^	0.414^**^	0.428^***^	0.451^***^	0.486^***^
	(3.832)	(4.482)	(2.458)	(4.847)	(4.994)	(5.089)
Public transfer income	0.114	0.103	0.101	0.085	0.097	0.076
	(1.483)	(1.256)	(1.028)	(0.907)	(1.105)	(0.901)
Household registration	0.281^***^	0.216^**^	0.256^**^	0.216^*^	0.216^**^	0.216^**^
	(2.915)	(2.101)	(2.484)	(1.681)	(2.478)	(2.365)
Educational attainment	0.092	0.074	0.085	0.075	0.082	0.083
	(1.244)	(1.002)	(1.018)	(0.874)	(0.912)	(1.028)
Number of children	0.043	0.042	0.036	0.051	0.046	0.062
	(0.564)	(0.495)	(0.387)	(0.725)	(0.601)	(0.675)
Health status	−0.436^***^	−0.427^**^	−0.504^*^	0.483^**^	0.451^**^	0.505^*^
	(−3.542)	(−2.428)	(−1.165)	(2.271)	(2.453)	(1.848)
Pension insurance satisfaction	0.365^***^	0.407^***^	0.412	0.408^***^	0.486^**^	0.496
	(3.748)	(3.852)	(1.401)	(3.924)	(2.185)	(1.377)
Satisfaction with healthcare insurance	0.247^***^	0.253^**^	0.314	0.328^***^	0.315^**^	0.486
	(3.621)	(2.345)	(1.504)	(3.617)	(2.265)	(1.408)
Constant term (math.)	22.145	25.471	28.047	22.847	26.065	24.431
*F*-value	155.931	193.647	201.512	147.841	158.577	198.301

^*^p < 0.1, ^**^p < 0.05, ^***^p < 0.01.

The results show that the Gini coefficient has a significant negative impact on the state of mind of older adults, indicating that worsening income inequality greatly affects their psychological health. Additionally, the state of mind of older adults significantly impacts the utilization of therapeutic and preventive healthcare. This demonstrates that the impact of income inequality on the utilization of these healthcare services can be mediated by the psychological state of older adults, thus verifying research hypothesis C (test in Table 6).

In order to verify whether the public transfer income can alleviate the negative impact of income inequality on the utilization of healthcare services, the product term of Gini coefficient and public transfer income is introduced into the baseline model, and the estimation results in Table 7 show that, in the full-sample regression, the coefficients of the product term of the Gini coefficient and the public transfer income are all significantly positive in the model (1)-model (6), and public transfer income plays a positive moderating effect in the process of the influence of income inequality on the utilization of healthcare services for the older adults. The public transfer income has the greatest moderating effect on the utilization of curative and preventive healthcare services by the low-income older adults, and the smallest moderating effect on the high-income older adults (test in Table 7). Table 7 shows mediated effects test.

**Table 7:** Moderating effects test.

	**Therapeutic services**			**Preventive healthcare services**		
	**Lower (1)**	**Medium (2)**	**High (3)**	**Lower (4)**	**Medium (5)**	**High (6)**
Gini coefficient X public transfer income	0.821^***^	0.764^*^	0.258^*^	0.706^**^	0.833^***^	0.216^*^
	(5.324)	(1.742)	(1.878)	(2.098)	(4.706)	(1.774)
Gini coefficient	−0.563^***^	−0.474^**^	−0.346^**^	−0.254^***^	−0.548^**^	−0.367^***^
	(−3.447)	(−2.461)	(−2.404)	(−3.649)	(−2.428)	(−3.741)
Public transfer income	0.651^***^	0.645^***^	0.552^***^	0.534^***^	0.649^***^	0.605^***^
	(4.541)	(4.785)	(4.247)	(6.354)	(6.047)	(5.782)
Control variables	Yes	Yes	Yes	Yes	Yes	Yes
*P*-value for the DWH test	0.031	0.038	0.021	0.011	0.004	0.012
Cragg-Donald statistic	221.038	203.541	183.479	147.654	132.286	121.358

^*^p < 0.1, ^**^p < 0.05, ^***^p < 0.01.

## Robustness test

In this study, we choose “healthcare services expenditure” and “healthcare services quality” to replace the dependent variables in the above study for the robustness test. The specific questions are “How much did you spend on healthcare services in the past year” and “What do you think about the current level of healthcare services.” Considering that the model setting and other issues may bring some bias, this study uses the Probit model instead of the above regression model for robustness testing. The results of the Probit model are shown in Table 8. Income inequality has a negative impact on both healthcare spending and health care quality of the older adults. This is consistent with the results of the analysis above, indicating that the previous analysis is the conclusion is documented reliable.

**Table 8:** Robustness test.

	**Healthcare services expenditure**		**Healthcare services quality**	
	**Model 5**	**Model 6**	**Model 7**	**Model 8**
Gini coefficient	−0.216^***^	−0.365^***^	−0.448^**^	−0.516^***^
	(−3.041)	(−3.228)	(−2.481)	(−4.458)
Household income		0.209^***^		0.147^***^
		(3.125)		(3.675)
Public transfer income		0.269^***^		0.412^*^
		(3.449)		(1.852)
Control variables	Yes	Yes	Yes	Yes
LR/Wald test	441.510^***^	516.221^***^	385.750^***^	462.175^***^

^*^p < 0.1, ^**^p < 0.05, ^***^p < 0.01.

## Discussion

The study's findings indicate that low-income older adults are more likely to experience a correlation between income inequality and access to curative and preventive healthcare services. In contrast, high-income older adults do not appear to be as significantly affected by this relationship. This emerging trend suggests that income inequality may be a key factor influencing healthcare services, particularly among low-income individuals. The underlying causes of this phenomenon may be attributed to several factors, including the impact of economic stability. Individuals in the older adults bracket who are in receipt of higher incomes are likely to have a more stable financial situation and savings, which means that they are less likely to be affected by minor fluctuations in income or income inequality within society (45, 46). Individuals in the highest income brackets are more likely to have comprehensive health insurance coverage. This may result in a reduced impact of direct financial pressures on healthcare utilization, as the majority of costs may already be covered by insurance companies (47). Higher-income older adults are likely to place more emphasis on their health and have regular healthcare checkups compared to the rest of the population (48). It is possible that high-income older adults may not require any changes to their preventive healthcare behaviors as a result of income inequality. Finally, the influence of culture and values must be considered. High-income older adults may prioritize long-term health and quality of life, with the majority espousing the view that health should not be compromised even in the context of societal inequality (49).

The results of the study indicate that the impact of pension insurance satisfaction and health insurance fullness on the utilization of healthcare services varies for older adults with different income levels. The greatest impact is observed in medium-income older adults. The reasons behind this are mainly due to the following factors: firstly, the effect of financial pressure. Low-income older adults may face more financial pressure, and even with pension insurance, they may still be skeptical or avoid using healthcare services due to the pressure of other living costs (50). Higher-income older adults may be less reliant on insurance satisfaction due to their relative financial wellbeing, as they can afford to pay more for healthcare. In contrast, medium-income older adults value insurance satisfaction highly because it is directly related to whether they can access the healthcare services they need (51). Secondly, the influence of expectations. Middle-income older adults tend to have certain expectations regarding their health status and healthcare services. They may desire access to high-quality healthcare services, yet are reluctant to spend excessively. Consequently, when they are content with their pension insurance, they are more likely to utilize healthcare services (52). Thirdly, the influence of information access and decision-making on health outcomes must be considered. Individuals in higher income brackets may have greater access to healthcare resources and information, including private medical practitioners and a wider range of healthcare options (53). Low-income older adults may rely more on public healthcare services. Medium -income older adults may rely more on information and services provided by pension insurance (54), so their satisfaction with pension insurance can directly affect their healthcare decisions. Finally, social support and cultural factors. Medium -income older adults may have broader social support and social networks that encourage and support their active participation in preventive healthcare (55).

It has also been found that the impact of health insurance and pension insurance satisfaction on healthcare service utilization varies across regions. The impact of health insurance and pension insurance satisfaction on healthcare service utilization is found to be insignificant in the eastern region and significant in the central and western regions. The greatest impact is observed in the central region. This is attributed to the level of economic development, with eastern regions typically experiencing faster economic growth and having a greater abundance of both public and private healthcare resources compared to the western and central regions (56). Therefore, even if older adults in the eastern region are not satisfied with pension and health insurance, they can still obtain healthcare services through other means. The economic situation in the central region may make it difficult for many older adults to afford the high cost of healthcare, while pension and health insurance provide a more economically viable option for them. Secondly, it is possible that healthcare resources in the central and western regions may be less abundant than in the eastern regions. Consequently, satisfaction with pension insurance and healthcare insurance may be more important in these regions (57). Finally, demographic factors must be considered. The eastern region may have attracted a large number of young laborers due to earlier economic development, while the central and western regions may have more aging problems, affecting the demand and reliance on healthcare services by older adults in each region (58). In short, a combination of factors may have made the effect of pension insurance satisfaction on the utilization of healthcare services by older adults (59).

Additionally, there are notable variations in the influence of public transfer revenues on healthcare utilization across regions. In the eastern region, the impact of public transfer revenues on healthcare utilization is relatively insignificant, whereas in the central and western regions, the impact is considerably more pronounced, with the greatest impact observed in the western region. Potential explanations for this discrepancy may include differing economic conditions between the western and eastern regions. In the eastern regions, it is possible that average income levels or more affluent areas may be higher, which could enable older adults to more readily pay for healthcare. Consequently, the impact of public transfer revenues on healthcare delivery may be more pronounced in these regions (60, 61). The western region may have more policy initiatives to encourage older adults to receive healthcare services, making public transfers more influential.

The impact of income inequality on the psychological health of older adults and its subsequent effect on healthcare utilization is a complex, interrelated process. According to social comparison theory, which suggests that people tend to compare themselves to others in their social environment, income inequality has a negative and significant impact on older adults' psychological health and inhibits their healthcare utilization (32).

The observation of economic disparities between oneself and others may result in psychological dissatisfaction, frustration, and low self-esteem, which can have a negative impact on psychological health. Concurrently, increased income inequality can lead to increased financial stress and insecurity among older adults. This financial stress may result in chronic psychological stress, thereby increasing the risk of psychological health problems. Self-efficacy theory posits that older adults who experience economic deprivation may exhibit lower self-efficacy, which in turn may reduce the likelihood that they will actively seek out healthcare services. Social inequality may lead to lower social support for low-income older adults, which may further affect their psychological health (62, 63). Psychological health issues may result in a reluctance to seek healthcare services, which in turn may lead to a reduction in service utilization. In summary, income inequality has a detrimental impact on the psychological wellbeing of older adults, which in turn affects their likelihood and manner of utilizing healthcare services. This interrelated process underscores the significance of ensuring equitable and comprehensive healthcare for older adults and the urgency of addressing the issue of income inequality.

The study finds that public transfer income reduces healthcare inequality among older adults, with the strongest positive impact on low-income individuals and the weakest on high-income individuals. The reasons include public transfers acting as a critical income source for low-income older adults, making them more likely to use healthcare services, and their greater sensitivity to healthcare spending changes. In contrast, higher-income older adults, who may have other resources or private insurance, are less affected by public transfers in their healthcare utilization choices.

Low-income older adults may have previously neglected preventive healthcare services due to financial constraints. However, with the support of public transfer income, they are more likely to take preventive measures, thus reducing their future healthcare expenditures. Another possible reason is the setting of policy goals. Public policies in many countries aim to reduce poverty and social inequality. As a result, the design of public transfer revenues may deliberately favor helping those most in need, namely low-income older adults. Public transfer revenues serve as an economic safety net, playing an important role in reducing inequalities in healthcare utilization among older adults. For low-income older adults, such support is particularly critical to help them access necessary healthcare services.

## Innovation

The findings indicate that public transfer income plays a moderating role, with a significant “reordering effect” on income inequality and a “deprivation effect” on income inequality. This, in turn, inhibits healthcare utilization among older adults by affecting their psychological state. First, the study clearly identifies a significant negative relationship between income inequality and healthcare utilization among older adults, with this relationship being particularly pronounced among older adults in low- and medium-income and Western regions. This meticulous examination of geographic and income level heterogeneity is highly valuable in this field of research. Secondly, this study attempts to integrate the two key dimensions of public transfer income and psychological state, thereby revealing how they play a moderating and mediating role in this relationship. A new framework for understanding is provided. Moreover, the study offers a more comprehensive and nuanced perspective on the relationship between income inequality and healthcare utilization among older adults through in-depth analysis of empirical data from three major geographic regions. These distinctive research perspectives and in-depth analyses distinguish this study from others in the field of healthcare services for older adults and provide valuable references and insights for subsequent studies.

## Conclusions

The rapid growth of China's older adult population has led to a corresponding increase in their healthcare needs and service utilization patterns, which have become increasingly prominent in the socio-economic structure. This study aims to provide insights into how income inequality affects the utilization of healthcare services by older adults, and to further examine the moderating roles of public transfer income and the psychological health of older adults in this process.

The impact of income inequality and its heterogeneity on the utilization of healthcare services by older adults is a significant area of study. The Gini coefficient has been found to have a negative correlation with the utilization of healthcare services by older adults, with this correlation being more pronounced among older adults in low- and medium-income and western regions. In contrast, older adults in high-income and eastern regions were relatively insensitive to the impact of income inequality. Public transfers play a moderating role, with a significant “reordering effect” on income inequality. They have a significant positive moderating effect on healthcare utilization among low- and medium-income older adults and those in the West, but not among high-income older adults and those in the East. Public transfers had the largest moderating effect on the utilization of curative and preventive healthcare services among low-income older adults, and the smallest moderating effect among high-income older adults. The mediating effect of psychological health on the relationship between income inequality and healthcare utilization among older adults is evident. Income inequality may have a negative impact on the psychological health of older adults, which in turn reduces their willingness to utilize healthcare services and their frequency of utilization.

This study identifies the negative impact of income inequality on the utilization of healthcare services by older adults and highlights the moderating role of public transfer income and older adults' psychological health in this process. Considering the heterogeneity among different income and regional groups, policymakers and relevant institutions should develop differentiated strategies based on different group characteristics to ensure that older adults have equitable access to healthcare services.

## Contributions to the literature

This study demonstrates a significant negative relationship between income inequality and healthcare utilization among older adults, especially in low- and medium-income groups and in western regions.

It identifies the mediating role of psychological health, revealing that income inequality can lead to negative psychological effects, reducing healthcare utilization among older adults.

The study explores the moderating role of public transfer income, particularly for low-income older adults, by mitigating the negative effects of income inequality.

By categorizing therapeutic and preventive healthcare service utilization, the study provides valuable insights for addressing disparities in healthcare utilization due to income inequality.

## Data availability statement

The original contributions presented in the study are included in the article/supplementary material, further inquiries can be directed to the corresponding authors.

## Ethics statement

Ethical approval for this study was granted by the Medical Ethics Committee of Xi'an Jiaotong University Health Sciences Center (Approval No. 2018-1200). The studies were conducted in accordance with the local legislation and institutional requirements. The participants provided their written informed consent to participate in this study.

## Author contributions

ZC: Conceptualization, Data curation, Formal analysis, Funding acquisition, Investigation, Software, Writing – original draft, Writing – review & editing. HL: Formal analysis, Writing – review & editing, Data curation, Investigation, Software. JM: Formal analysis, Writing – review & editing, Conceptualization, Funding acquisition, Resources, Supervision, Visualization, Writing – original draft. YZ: Conceptualization, Funding acquisition, Investigation, Resources, Software, Validation, Visualization, Writing – original draft, Writing – review & editing.
